# Fermentation as a Tool to Revitalise Brewers’ Spent Grain and Elevate Techno-Functional Properties and Nutritional Value in High Fibre Bread

**DOI:** 10.3390/foods10071639

**Published:** 2021-07-15

**Authors:** Emma Neylon, Elke K. Arendt, Emanuele Zannini, Aylin W. Sahin

**Affiliations:** 1 School of Food and Nutritional Science, University College Cork, College Road, T12K8AF Cork, Ireland; emma.neylon@umail.ucc.ie (E.N.); e.zannini@ucc.ie (E.Z.); aylin.sahin@ucc.ie (A.W.S.); 2 APC Microbiome Ireland, University College Cork, Western Road, T12K8AF Cork, Ireland

**Keywords:** fibre, fermentation, wheat bread, by-product utilisation, brewers’ spent grain

## Abstract

Recycling of by-products from the food industry has become a central part of research to help create a more sustainable future. Brewers’ spent grain is one of the main side-streams of the brewing industry, rich in protein and fibre. Its inclusion in bread, however, has been challenging and requires additional processing. Fermentation represents a promising tool to elevate ingredient functionality and improve bread quality. Wheat bread was fortified with spray-dried brewers’ spent grain (BSG) and fermented brewers’ spent grain (FBSG) at two addition levels to achieve “source of fibre” and “high in fibre” claims according to EU regulations. The impact of BSG and FBSG on bread dough, final bread quality and nutritional value was investigated and compared to baker’s flour (BF) and wholemeal flour (WMF) breads. The inclusion of BSG and FBSG resulted in a stronger and faster gluten development; reduced starch pasting capacity; and increased dough resistance/stiffness. However, fermentation improved bread characteristics resulting in increased specific volume, reduced crumb hardness and restricted microbial growth rate over time. Additionally, the inclusion of FBSG slowed the release in reducing sugars over time during in vitro starch digestion. Thus, fermentation of BSG can ameliorate bread techno-functional properties and improve nutritional quality of breads.

## 1. Introduction

Brewers’ spent grain (BSG) has been labelled as the most abundant side-stream generated by the brewing industry and accounts for approximately 85% of the total by-products produced [1]. BSG is a lignocellulosic material comprised of the outer layers of the barley grain, namely the husk-pericarp-seed coat [1]. It is rich in dietary fibre (30–50%), mainly arabinoxylan, and protein (19–30%), contains low levels of fat and starch, as well as vitamins and minerals [2]. However, variations in the composition of BSG are common [2,3], which can be associated with numerous factors such differences in barley variety, harvesting conditions, malt type, the adapted brewing process and the addition of adjuncts during brewing, the point at which BSG is retrieved from the brewing process and also where in the filter cake the BSG sample is retrieved [1,3,4,5]. The primary outputs for BSG are in animal nutrition and landfill. However, numerous alternative uses for BSG are emerging [1,2,3,6,7]. As BSG is considered a highly nutritious raw material, increased attention has been given to the use of BSG as an ingredient in different food products to elevate their nutritional value and pursue the goal of a more sustainable future.

The demand for high fibre foods has increased due to consumers’ awareness of the health benefits associated with the intake of dietary fibre, such as reducing the risk of cardiovascular disease [8], lowering cholesterol [9] and preventing the development of colorectal cancer [10]. Even though the demand for foods rich in dietary fibre has increased, most people following a Western diet fail to meet the recommended daily intake of 25 g fibre [11]. With fibre holding a significant proportion of BSG composition, its use as a fibre fortifier in food in the future is of high interest. Previous studies implementing BSG into food such as bread [12,13,14,15,16], pasta [17,18], breadsticks [19], crisp-slices [20], cookies [21], extruded snacks [22,23] and meat [24] have shown promising outcomes with increasing fibre contents of foods. Although BSG elevates the nutritional value of the food products, it affects the techno-functional characteristics of the systems, particularly in bread, leading to a poor-quality bread with respect to the specific volume and crumb texture [12,16]. However, great successes have been observed in relation to nutritional and techno-functional properties of both bread and pasta with the inclusion of two refined BSG ingredients, EverVita Fibra and EverVita Pro, which highlights the potential of BSG as a food ingredient after additional processing [25,26].

Fermentation with lactic acid bacteria (LAB) has proven to be a valuable tool in compensating for quality loss of bread systems in various studies, leading to positively enhancing some technological aspects, such as extending microbial shelf life [27], improving dough quality [28], reducing staling [12,29] and increasing specific volume [29]. In addition to the technological benefits, improved flavour profiles [30], reduced glycaemic responses [31], and enhancements in antioxidant activity [32] have also been observed. Previous studies incorporating BSG in bread formulations in the form of sourdough fermentation reported a positive impact on bread crumb hardness [12,16], an increase in bread specific volume [16] and also an alteration of the sensory profile of the breads [12,14]. Hence, fermentation technology represents a promising approach to overcome quality losses in cereal-based products fortified with BSG. In a previous study, the incorporation of fermented BSG in pasta showed superior product quality regarding technological and nutritional characteristics compared to wholemeal pasta (Neylon et al.; submitted for publication).

The current study reveals the effect of partial replacement of wheat flour with spray-dried BSG (BSG) and spray-dried fermented BSG (FBSG) on dough rheology and techno-functional, structural, and nutritional characteristics of bread. BSG and FBSG were included in bread formulations in two different concentrations to achieve “source of fibre” and “high in fibre” health claims according to EU Regulation (EC) No 1924/2006 [33]. Baker’s wheat flour (BF) and wholemeal flour (WMF) were used as controls throughout the study.

## 2. Materials and Methods

### 2.1. Raw Materials

Flour ingredients incorporated in bread recipes include: baker’s flour (BF) supplied by Odlums Group, Dublin, Ireland; stone ground wholemeal flour (WMF) from Odlums Group, Dublin, Ireland; milled and spray-dried brewers’ spent grain (BSG); and milled and spray-dried fermented brewers’ spent grain (FBSG). BSG and FBSG ingredients were provided by Anheuser-Busch InBev SA/NV (Leuven, Belgium). FBSG was produced using a patented process (patent number: WO 2018/033521 A1) [34] using a combined saccharification and fermentation process using lactic acid bacteria *Lactobacillus plantarum* f10 and/or *Lactobacillus rhamnosus* GG (LGG^®^). Compositional analysis of ingredients used in the study are provided on Table 1. The WMF, BSG and FBSG composition in Table 1 were reported in a previous study (Neylon et al.; submitted for publication). BF compositional analysis was completed by an accredited laboratory (Concept Life Sciences Ltd., Bar Hill, UK). Alpha-amylase activity was determined in duplicate using the alpha-amylase assay kit (ceralpha method) supplied by Megazyme (Bray, Co. Wicklow, Ireland). Other ingredients used in bread recipes include instant active dried baker’s yeast *Saccharomyces cerevisiae* (Puratos, Groot-Bijgaarden, Belgium), sugar (Siúcra, Dublin, Ireland), salt (Glacia British Salt Limited, Cheshire, UK), sunflower oil (Musgraves, Cork, Ireland) and tap water. All chemicals used in experiments were purchased from Sigma Aldrich (St Louis, MO, USA). The addition of BSG and FBSG was adjusted in accordance with “source of fibre” (SF) and “high in fibre” (HF) claims [33]. This claim applies to the final food product, implying the final food product contains 3 g/100 g fibre (SF) and 6 g/100 g fibre (HF).

### 2.2. Dough Analysis

#### 2.2.1. Water Content Adjustment

Farinograph-TS^®^ (Brabender GmbH and Co KG, Duisburg, Germany), equipped with an automatic water dosing system (Aqua inject), was used to determine the water addition level of the different formulations. The target consistency was set to 500 ± 20 FU and the temperature of the kneading chamber was 30 °C. The formulations included the controls BF and WMF, as well as the blends of BF and BSG/FBSG in the proportions demonstrated in Table 2.

#### 2.2.2. Gluten Aggregation Analysis

GlutoPeak (Brabender GmbH and Co KG, Duisburg, Germany) was used to determine the quality of the gluten network. Before analysis, flour blends according to the flour/ingredient proportions given in Table 2 were prepared. Flour blends were premixed to ensure homogeneity before analysis. Nine grams of sample (based on 14% moisture) was added to deionised water (36 °C) to a total volume of 18 g, and the test was started using a shear speed of 2750 rpm. The chamber temperature was set to 36 °C. Torque was monitored over time (s). The torque maximum (TM) in Brabender Units (BU), and the peak maximum time (PMT) in seconds (s) were evaluated.

#### 2.2.3. Starch Pasting Properties

Starch pasting behaviour was analysed using a Rapid Visco Analyser (RVA) (RVA Super 3, Newport Scientific, Warriewood, Australia). Therefore, blends of BF and BSG/FBSG were prepared according to the inclusion level illustrated in Table 2. Three grams of sample (based on 14% moisture) was added to 25 g of deionised water in an aluminium cup. Before the test, the sample was dispersed in the water using the RVA-paddle. A temperature profile was applied under constant shear of 160 rpm, starting at 50 °C for 60 s, followed by heating to 95 °C at a heating rate of 0.2 °C/s, holding the temperature for 162 s, cooling to 50 °C at a cooling rate of 0.2 °C/s and holding the final temperature for 120 s. Parameters analysed included peak viscosity, breakdown viscosity, trough viscosity and final viscosity in centipoise (cP).

#### 2.2.4. Bread Dough Preparation

Bread doughs were produced by mixing the dry ingredients first, followed by the addition of yeast solution and sunflower oil. Yeast solution was prepared by adding instant active dried yeast in water (25 °C) and allowing to activate for 10 min. After the addition of the liquids, the ingredients were mixed (MACPAN MX 10 spiral mixer, MACPAN SNC, Thiene, Italy) at speed 1 for 6.5 min, followed by a second stage of mixing at speed 2 for 5 min.

#### 2.2.5. Evaluation of Bread Fermentation Quality

The fermentation quality of each bread dough was analysed using a Rheofermentometer (Chopin, Villeneuve-la-Garenne CEDEX, France). Three hundred grams of bread dough (prepared according to Section 2.2.4.) was placed into the fermentation chamber and a 1500 g cylindrical weight was placed on top of the dough. The chamber was closed, and the dough was left to ferment for 3 h at 30 °C. The maximum dough height (Hm) in mm, volume of CO_2_ produced during fermentation in ml and CO_2_ retention coefficient in % was evaluated.

#### 2.2.6. Dough Rheology

Viscoelastic properties of doughs (prepared according to Section 2.2.4) were analysed using a Rheometer Physica MCR 301 (Anton Paar GmBH, Ostfildern, Germany). Yeast was omitted. Serrated plates were placed in parallel geometry. The lower plate was held at 35 °C throughout the analysis, accompanied by an upper plate of 50 mm in diameter. Dough samples were loaded onto plates and the linear viscoelastic region was determined using an amplitude sweep as described by Hager et al. [36]. Frequency sweeps were performed using a constant strain of 0.01% and a frequency range from 100 to 0.1 Hz (data obtained at angular frequency 2.58 Hz). Prior to analysis, dough samples were left to rest for 5 min to allow for equilibration. The damping factor ($\tan\delta \;\frac{G''}{G'}$) was evaluated to investigate the extent of changes in viscoelastic properties of dough samples with fibre ingredient addition.

### 2.3. Bread Production

Bread dough was produced following Section 2.2.4. A total dough volume of 2500 g was divided into five 450 ± 1 g pieces, moulded, transferred to greased tins, and proofed in a proofing chamber (KOMA SunRiser, Roermond, The Netherlands) for 90 min at 35 °C and 75% humidity. After proofing, the bread loaves were baked in a deck oven (MIWE Condo, Arnstein, Germany) for 35 min at 220/230 °C top/bottom temperature. Before loading, 400 mL of steam was injected into the oven, leaving the draft open throughout baking. Following baking, bread loaves were left to stand for 1 h to cool before analysis. Each recipe outlined in Table 2 was baked and analysed in triplicate. Values reported represent the mean of three independent baking trials.

### 2.4. Bread Analysis

#### 2.4.1. Bake Loss

Bake loss was determined to investigate the amount of water lost due to baking. The bake loss of five bread loaves per batch was measured. This was calculated in percentage according to the following formulas:

(1) Weight of dough before baking (g)−Weight of bread after baking (g)=Moisture lost from bake (g)

(2) $$\frac{\mathrm{Moisture}\;\mathrm{lost}\;\mathrm{from}\;\mathrm{bake}\;\left(g\right)}{\mathrm{Weight}\;\mathrm{of}\;\mathrm{dough}\;\mathrm{before}\;\mathrm{baking}\;\left(g\right)}\times 100=\mathrm{Bake}\;\mathrm{loss}\;\%$$

#### 2.4.2. Specific Volume

Specific volume was calculated using a Volscan Profiler (Stable Micro Systems, Surrey, UK), measured in mL/g. Two loaves per batch were analysed.

#### 2.4.3. Crumb Structure

Analysis of bread crumb structure was completed using a C-Cell Imaging System (Calibre Control International Ltd., Warrington, UK). Two loaves per batch were sliced into 25 mm thick slices. The crust slices were omitted from the analysis and only centre slices (five per loaf) were considered. The C-Cell Imaging System was used to provide numerical data on slice area, number of cells and cell diameter.

#### 2.4.4. Texture

Crumb texture was determined using a TA-XT2i Texture Analyser (Stable Micro Systems, Surrey, UK) equipped with a 25 kg load cell. A two-compression test with a strain of 40%, test speed of 5 mm/s, a trigger force of 0.05 N and a waiting time of 5 s between the two compressions was chosen. A 35 mm cylindrical probe was used in the analysis. Bread slices with a thickness of 25 mm were measured and the crumb hardness and the crumb resilience was analysed on the day of baking. Crumb hardness was calculated as the maximum force of the 1st compression and crumb resilience was calculated by dividing the upstroke energy of the first compression by the down stroke energy of the first compression. Bread staling was determined by measuring the crumb hardness over five days. The staling rate was determined as reported by Sahin et al. [37].

#### 2.4.5. Colour

Crumb and crust colour were determined using a hand-held colorimeter (Minolta CR-331, Konica Minolta Holdings Inc., Osaka, Japan). The CIE L*a*b* colour system was used for colour evaluation. The differential colour index (ΔE) was determined according to the below equation to evaluate the changes in the colour of the crust and crumb with BSG and FBSG inclusion.

(3) $$\Delta E=\sqrt{{\left({\Delta L}^{*}\right)}^{2}+{\left({\Delta a}^{*}\right)}^{2}+{\left({\Delta b}^{*}\right)}^{2}}$$

where ${\Delta L}^{*}$ = L*_control_ − L*_sample_, Δa* = a*_control_ − a*_sample_ and ${\Delta b}^{*}$ = b*_control_ − b*_sample_.

#### 2.4.6. Water Activity and Microbial Shelf Life

Water activity was determined using the water activity meter AquaLab series 3 (Decagon Devices Inc., Pullman, WA, USA). The influence of fibre ingredients on microbial shelf life was analysed using the mould environmental challenge method indicated by Dal Bello et al. [29] and Sahin et al. [38] with slight modifications. Briefly, ten centre slices of 25 mm thickness (two bread loaves) per batch were placed on a sterile metal rack. The bread crumb of both sides of the bread was exposed to the environment for 5 min. Bread slices were packed singly in sterile bags and heat-sealed. A filter pipette was placed in each bag to allow for consistent aerobic conditions to prevail. Bread samples were stored at 20 ± 1 °C and 50% relative humidity in a sterilised and temperature-controlled chamber (KOMA SunRiser, Roermond, The Netherlands) for 14 days. Mould growth of each bread slice was visually analysed daily, and mould growth was rated as “mould free”, “mould growth <10%”, “10–24% mould growth”, “25–49% mould growth” and “mould growth >50%”.

#### 2.4.7. In Vitro Starch Digestibility

An in vitro digestion assay based on enzymatic degradation of starch to reducing sugars over time designed for fibre enriched products was conducted as reported by Brennan and Tudorica [39]. Briefly, 4 g of crushed bread samples were exposed to a 30 min proteolytic treatment using pepsin solution. After this, samples were placed in 1-inch width dialysis tubing, suspended in sodium potassium phosphate buffer (pH 6.9) and incubated for 5 h with a pancreatic α-amylase solution. Samples were taken every 30 min and dialysis tubing were inverted every 15 min. To determine the amount of reducing sugars (maltose) released over time spectrophotometrically (wavelength 546 nm), 100 µL of the samples taken were diluted with 100 µL of 3,5-dinitrosalicyclic acid, heated to 100 °C for 15 min and then diluted with 1 mL of deionised water. Analysis was completed in duplicate. Reducing Sugar Release % (RSR%) overtime was calculated according to Brennan and Tudorica [39] using the following formula:

(4) $$\mathrm{RSR}\%=\frac{A_{\mathrm{sample}}\times 500\times 0.95}{A_{\mathrm{maltose}}\times \mathrm{available}\;\mathrm{carbohydrate}\;}\times 100$$

where A_sample_ represents the active sample absorbance at 546 nm; 500 (mL) displays the solution total volume; 0.95 is the maltose to starch conversion factor, A_maltose_ indicates the absorbance of 1 mg of pure maltose/mL buffer; and available carbohydrate (in mg) represents sugar and digestible starch present in 4 g of sample. Available carbohydrate values were determined using the digestible carbohydrate values measured using the Megazyme kit K-RAPRS (Bray, Ireland).

The starch digestibility is presented as the release of reducing sugars over time in the form of a plot. The slope of the curves was calculated using Microsoft Excel after ensuring a linearity (r^2^ > 0.99).

#### 2.4.8. Bread Microstructure

Bread samples were freeze-dried, mounted on stubs (G 306; 10 mm × 10 mm Diameter; Agar Scientific, Stansted, UK) and fixed using carbon tape (G3357N; Carbon Tabs 9 mm; Agar Scientific, Stansted UK). Mounted bread samples were sputter-coated with a gold-palladium alloy (ratio of 80: 20), using a Polaron E5150 sputter coating unit, and imaging was captured with a JEOL Scanning Electron Microscope (JSM-5510, Jeol Ltd., Tokyo, Japan). Settings for analysis were as follows: 5 kV 185 voltage, 20 mm working distance and a magnification factor of 1000.

### 2.5. Statistical Analysis

Analysis was conducted in triplicate unless stated otherwise. A one-way ANOVA with post hoc pairwise Tukey test (*p* value ≤ 0.05) was performed using statistical software SPSS to determine significant differences between groups. When equal variances were not assumed, a correction using welch test and Games-Howell post hoc test (*p* ≤ 0.05) was applied. A two-way ANOVA was conducted to evaluate the effect of the type of ingredient and addition level on parameters using Statistical software Minitab version 19 (Minitab Inc., State College, PA, USA). Correlation analysis was carried out using Microsoft Excel.

## 3. Results

### 3.1. Dough Analysis

#### 3.1.1. Water Absorption

Determining the optimal water addition and dough consistency of the dough is necessary to ensure optimal conditions for dough hydration and gluten network formation. Results from farinograph water absorption (FWA) analysis are displayed in Table 3.

Significant differences in FWA capacities occurred between the controls BF (57.30 ± 0.2%) and WMF (59.33 ± 0.15%). Furthermore, the replacement of BF by BSG and FBSG caused an increase in water absorption, particularly in high in fibre formulations (BSG HF (68.60 ± 0.35%); FBSG HF (66.93 ± 0.31%)). BSG HF showed the highest water absorption among all samples.

#### 3.1.2. Gluten Network Formation

**Figure 1:**
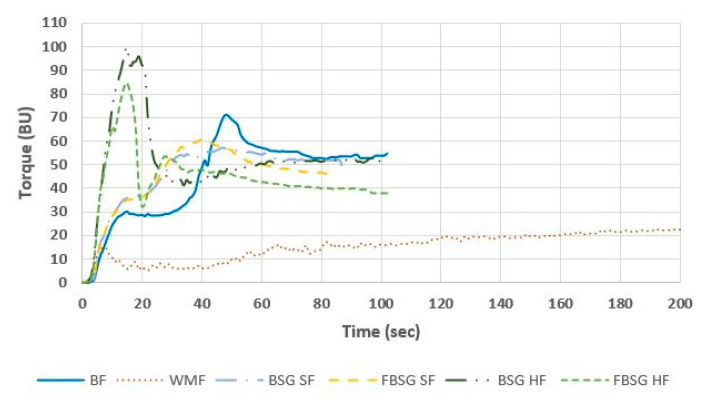
Graphical representation of results from GlutoPeak analysis using baker’s flour (BF), wholemeal flour (WMF), brewers’ spent grain (BSG) and fermented brewers’ spent grain (FBSG). SF and HF denotes “source of fibre addition level” and “high in fibre addition level”, respectively.

Ingredients rich in dietary fibre influence the gluten network formation. The gluten network development time (PMT) and the torque maximum (TM) of the different formulations are illustrated in Table 3. In addition, Figure 1 graphically displays the effect of BSG and FBSG inclusion on the network development.

BF showed a typical wheat flour gluten aggregation curve with a PMT of 48.67 ± 1.53 s and a TM of 71.67 ± 0.58 BU. In comparison, WMF displayed a prolonged increase in torque with a PMT of 141.33 ± 15.18 s and a weak gluten network (TM = 29.0 ± 1.0 BU).

The incorporation of BSG and FBSG in source of fibre levels weakened the gluten network significantly, resulting in TM of 57.33 ± 0.58 BU and 60.67 ± 0.58 BU in BSG SF and FBSG SF formulations, respectively. However, SF formulations showed stronger gluten aggregation than WMF. A faster gluten network formation occurred in BSG SF (47.67 ± 1.15 s) and FBSG SF (40.33 ± 0.58 s) recipes compared to BF.

Inclusion of BSG and FBSG at the HF level resulted in gluten aggregation curves that were not aligned with BF or WMF (Figure 1). Such formulations caused a rapid protein network formation followed by a fast breakdown. The replacement of BF by BSG in the high in fibre level showed the strongest gluten network (TM = 100.0 ± 2.0 BU), followed by FBSG HF (84.0 ± 2.65 BU). In addition, the high fibre formulations showed two peaks, indicating the aggregation of two protein groups. Furthermore, the network formation occurred at an earlier time point compared to BF (BSG HF = 15.67 ± 2.08 s; FBSG HF = 14.67 ± 0.58 s).

#### 3.1.3. Effect on Starch Behaviour with Fibre Ingredient Addition

The impact of BSG and FBSG in two inclusion levels on starch pasting properties are displayed in Table 3. The results show the peak viscosity (PV), final viscosity (FV), trough and breakdown viscosity (BV) of the different formulations.

The PV indicates the increase in viscosity during heating. The addition of fibre caused a decrease in PV. The highest PV occurred in BF (1007 ± 14.57 cP), while WMF caused the lowest PV (591 ± 30.85 cP). The incorporation of BSG and FBSG weakened the pasting behaviour of the system, resulting in a decrease in PV with the increase of addition level. Comparing BSG and FBSG with each other, FBSG showed a slightly lower peak viscosity at source of fibre levels (BSG SF (911.0 ± 15.13 cP), FBSG SF (900.66 ± 13.05 cP)), whereas BSG caused a lower PV at high in fibre levels ((BSG HF (701.33 ± 5.13 cP) and FBSG HF (760.67 ± 10.69 cP)).

The FV indicated the degree of retrogradation of the system after gelatinisation. BF and WMF showed the highest final viscosity with 1327.33 ± 26.58 cP and 1371.67 ± 27.61 cP, respectively. The addition of BSG or FBSG decreased the degree of retrogradation with increasing addition level. Comparing those two fibre ingredients with each other, FBSG caused a lower FV than BSG, particularly at the high in fibre addition level (BSG HF (1038.0 ± 11.79 cP); FBSG HF (643.0 ± 6.24 cP)).

Trough results reflect the viscosity of the suspension after the rupturing of the starch granules and indicates the system’s holding strength before the retrogradation process begins. The highest trough was noted in BF (607.0 ± 15.52 cP). Compared to BF, a reduction in trough viscosity was noted in WMF (486.0 ± 25.71 cP), BSG SF (543.67 ± 11.59) and FBSG SF (486.67 ± 13.58). The reduction in trough values was amplified at the HF addition level (BSG HF: 438.67 ± 3.79 cP, FBSG HF: 321.33 ± 10.26). Comparing the values obtained for BSG and FBSG, a greater reduction in trough viscosity was noted in FBSG formulations.

The breakdown viscosity represents the decrease in viscosity caused by the disruption of the gelatinised starch granules due to heat and shear after the peak viscosity has been reached. The highest BV was noted in FBSG HF (439.33 ± 7.23 cP), followed by FBSG SF (414.0 ± 3.61 cP) and BF (400.67 ± 1.15 cP). A reduction in BV was noted in the WMF (105.33 ± 10.96 cP) and BSG formulations (BSG SF: 367.33 ± 4.04 cP, BSG HF: 262.67 ± 1.53). Comparing BSG and FBSG formulations, a higher BV was observed for the FBSG formulations.

#### 3.1.4. Dough Rheology

The oscillatory damping factor (DF) indicates changes in the viscous and elastic proportions of the bread dough system. A system is defined as being an ideal elastic if the DF is 0, meaning no viscous parts are present. Hence, the higher the DF, the more viscous the system’s behaviour. The DF of the different formulations are illustrated in Table 3.

BF dough showed the highest DF (0.368 ± 0.015), indicating the highest viscous behaviour among all formulations, followed by WMF (0.341 ± 0.007). A significant reduction in DF values occurred with inclusion of BSG (SF: 0.331 ± 0.004; HF: 0.264 ± 0.005) or FBSG (SF: 0.330 ± 0.004; HF: 0.280 ± 0.003). Comparing both fibre ingredients with each other, the addition of BSG caused a greater shift towards elastic dough behaviour.

#### 3.1.5. Fermentation Capacity of Doughs

The fermentation capacity of the bread dough was determined using a Rheofermentometer and the results are demonstrated in Table 3.

Hm represents the maximum dough height achieved during dough fermentation. BF bread dough reached the highest dough height (53.33 ± 1.7 mm), whereas WMF bread dough resulted in a significantly lower Hm (20.3 ± 0.44 mm). The substitution of BF by BSG or FBSG resulted in a significant decrease in Hm with BSG SF and FBSG SF resulting in 35.03 ± 0.6 mm and 38.4 ± 3.48 mm, respectively. Comparing BSG and FBSG with each other, FBSG showed a slightly higher Hm at source of fibre addition level. No dough rise occurred in high in fibre BSG/FBSG formulations.

The volume of CO_2_ produced for BF dough was 2159.3 ± 132.03 mL. No significant differences in the volume of CO_2_ produced during fermentation were noted in WMF and BSG/FBSG formulations at either addition level compared to the BF control. The volume of CO_2_ produced during fermentation for these formulations was in the range of 2047.6–2228.3 mL (Table 3).

The CO_2_ retention coefficient represents the percentage of CO_2_ retained in the bread dough. BF dough had a CO_2_ retention coefficient of 98.73 ± 0.74%, while WMF dough had a slightly higher CO_2_ retention (99.60 ± 0.10%). No significant differences were noted in CO_2_ retention coefficients in comparison to BF for the BSG and FSBG ingredients, and were in the range of 99.20–99.73%.

### 3.2. Bread Analysis

#### 3.2.1. Bake Loss

Bake loss (BL) results are reported in Table 4. The highest bake loss was observed in BF (15.04 ± 0.53%), followed by FBSG (13.00 ± 0.69%) and BSG SF (12.89 ± 0.40%). An increased addition level of BSG and FBSG resulted in the lowest bake loss with 10.23 ± 0.26% and 10.47 ± 0.40%, respectively. BSG and FBSG affected the baking loss of the breads to the same extent.

#### 3.2.2. Specific Volume

Superior bread quality is often characterised by a bread with a high specific volume (SV). The results of the SV of the different bread formulations are illustrated in Table 4.

The SV for BF (5.49 ± 0.11 g/mL) was significantly higher than the SV recorded for the WMF (2.07 ± 0.11 g/mL). The inclusion of BSG and FBSG ingredients caused a decrease in SV of breads, with higher inclusion levels having more significant effects when compared to the BF control. However, in comparison to WMF, the SV of BSG SF (3.49 ± 0.13 g/mL) and FBSG SF (3.86 ± 0.21 g/mL) breads were significantly higher. The incorporation of BSG and FBSG in high in fibre concentrations resulted in the lowest SV with 1.45 ± 0.05 g/mL and 1.69 ± 0.03 g/mL recorded for BSG HF and FBSG HF, respectively. Comparing BSG and FBSG with each other, FBSG resulted in a higher SV.

#### 3.2.3. Crumb Structure

The crumb structure of the different bread formulations was investigated by the determination of the slice area, the number of cells, and the cell diameter. The results are presented in Table 4.

The biggest slice area occurred in the BF bread (11654 ± 361 mm^2^), followed by the source of fibre breads including FBSG (8788 ± 471 mm^2^) and BSG (8060 ± 313 mm^2^). The smallest slice area was detected in the breads including BSG and FBSG at high in fibre addition levels, with 4776 ± 278 mm^2^ and 5214 ± 234 mm^2^ reported, respectively.

Cells are created within the dough due to the production of CO_2_ during proofing. BF bread had the highest number of cells (6472 ± 282), while WMF bread showed the lowest number of cells (3250 ± 168). A significant decrease in the number of cells occurred in bread fortified with BSG or FBSG at both addition levels, which were, however, significantly higher than the WMF control.

**Figure 2:**
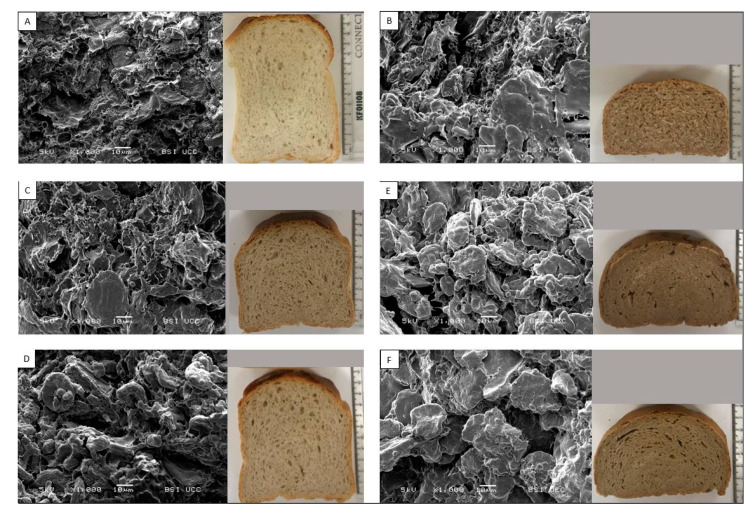
SEM micrographs of freeze-dried breads and images of their respective bread crumbs on day of baking. Pictures (**A**–**F**) illustrate baker’s flour (**A**), wholemeal flour (**B**), brewers’ spent grain “source of fibre” (**C**), fermented brewers’ spent grain “source of fibre” (**D**), brewers’ spent grain “high in fibre” (**E**) and fermented brewers’ spent grain “high in fibre” (**F**) breads.

BF had the largest cell diameter (2.28 ± 0.07 mm), followed by WMF (2.18 ± 0.14 mm). However, the result of WMF bread cannot be taken into account due to the imaging system potentially recognising larger bran particles as cells due to the dark colour. The WMF bread crumb image (Figure 2D) shows a dense crumb with limited gas cells embedded in the bread matrix versus BF. The inclusion of BSG and FBSG at the SF addition level reduced the cell diameter, resulting in 1.72 ± 0.08 mm and 1.86 ± 0.10 mm, respectively. The increase in addition level to HF amplified the reduction in cell diameter, leading to 1.09 ± 0.06 mm and 1.15 ± 0.07 mm in BSG HF and FBSG HF breads, respectively.

#### 3.2.4. Bread Texture and Staling

Crumb texture is considered an important parameter to analyse to ensure optimal bread quality. Values for crumb hardness, crumb resilience and the bread staling rate are presented in Table 4.

The softest crumb was determined in the BF bread (2.99 ± 0.36 N), while the WMF bread showed a significantly harder crumb (30.13 ± 6.15 N). The replacement of BF by BSG and FBSG at a source of fibre level increased crumb hardness to 10.91 ± 1.32 N and 7.91 ± 1.31 N, respectively. The increase in inclusion level of BSG and FBSG amplified the elevation in crumb hardness, resulting in the highest values (BSG HF: 79.22 ± 5.88 N; FBSG HF: 47.24 ± 3.97 N). Comparing BSG and FBSG with each other, FBSG caused a softer crumb.

The bread crumb with the highest resilience was found in the BF bread (0.49 ± 0.02 N), while a reduction in bread crumb resilience was observed in the WMF bread (0.41 ± 0.02 N). The inclusion of BSG and FBSG at the SF addition level further reduced the resilience of the crumb (0.46 ± 0.02 N and 0.47 ± 0.02 N, respectively). Increased levels of BSG and FBSG inclusion resulted in a greater decrease in crumb resilience (BSG HF: 0.34 ± 0.02 N, FBSG HF: 0.34 ± 0.02 N). No difference was observed between BSG and FBSG concerning the resilience of the bread crumb.

The staling of bread is the change in crumb hardness over time due to retrogradation and moisture migration. BF bread had the fastest staling rate (2.10 ± 0.49) while a reduction in the rate of staling occurred in WMF bread (1.0 ± 0.12). The replacement of BF with BSG and FBSG decreased the rate of staling to 1.34 ± 0.35 and 1.73 ± 0.13, respectively. Increased addition of BSG and FBSG resulted in an even lower staling rate (BSG HF: 0.70 ± 0.14, FBSG HF: 1.08 ± 0.06). When comparing staling results from the BSG and FBSG formulations, a slightly lower staling rate was observed in the BSG formulations; however, this was not statistically significant.

#### 3.2.5. Crust and Crumb Colour

Differences in the crust and crumb colour of breads were evaluated using ΔE values compared to BF and WMF bread, considering the differences in colour values L*, a* and b* compared to the controls.

Compared to BF, FBSG HF (11.99 ± 1.25) showed the greatest difference in crust colour, while BSG HF (9.26 ± 1.25), BSG SF (8.90 ± 1.03) and FBSG SF (8.39 ± 1.30) resulted in a more similar crust colour. Compared to WMF bread, a significant difference in crust colour was observed in BSG SF (20.91 ± 1.11) and FBSG SF (20.59 ± 1.07). The addition of higher amounts of BSG or FBSG caused a lower ΔE-value, however (BSG HF: 12.85 ± 0.96, FBSG HF: 10.52 ± 0.71).

The greatest difference in ΔE values for crumb colour compared to BF was observed in BSG HF (29.18 ± 0.55), followed by FBSG HF (28.31 ± 0.86). A reduction in ΔE crumb values occurred at the SF addition level (BSG SF:14.64 ± 1.16, FBSG SF 15.24 ± 0.95). In comparison to WMF crumb colour, the BSG HF had the highest ΔE (11.61 ± 0.77), followed by FBSG HF (10.50 ± 0.75), FBSG SF (9.17 ± 0.75) and BSG SF (8.54 ± 0.66).

#### 3.2.6. Water Activity and Microbial Shelf Life

The water activity (a_w_) of the bread crumb of the different formulations is illustrated in Table 4. BF (0.95 ± 0.01) and WMF (0.96 ± 0.007) bread crumbs exhibited a lower Aw than BSG and FBSG breads. The incorporation of BSG and FBSG led to an increase in Aw-value to 0.97 regardless the addition level or type of fibre ingredient.

**Figure 3:**
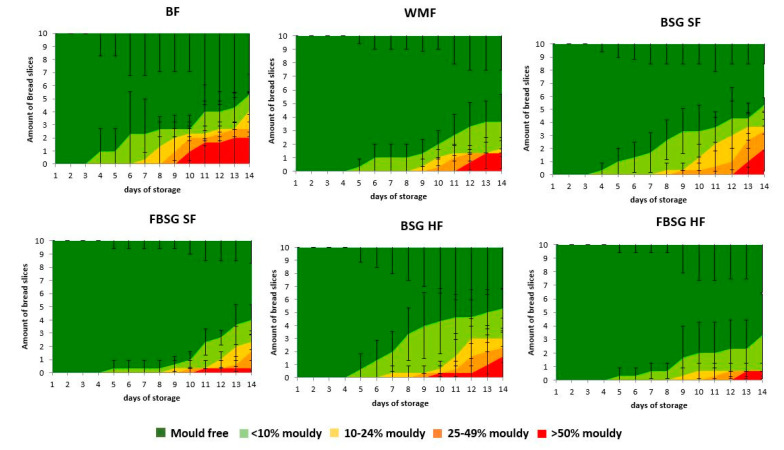
Shelf-life plots from 14-day analysis of breads. The amount of bread slices which contained each mould group (mould free, <10% mouldy, 10–24% mouldy, 25–49% mouldy and >50% mouldy) was counted over a period of 14 days. BF and WMF represent baker’s flour wheat control and wholemeal bread, respectively. BSG and FBSG denote brewers’ spent grain and fermented brewers’ spent grain breads, respectively. SF and HF stand for “source of fibre” and “high in fibre” addition levels, respectively. The graph represents mean values obtained across three independent batches with standard deviations included as error bars.

The microbial shelf life of the breads over time is demonstrated in Figure 3. The first mould growth on the BF bread occurred on day 4, while the shelf life of WMF bread was 5 days. The inclusion of BSG SF did not affect the microbial shelf life, whereas FBSG SF resulted in a prolonged shelf life by one day. Additionally, the inclusion of HF levels of both, BSG or FBSG, resulted in breads with a shelf life of 5 days. Even though the day of the first mould growth was very similar, the kinetics of mould growth were different, particularly when FBSG was used as a fibre ingredient. Figure 3 shows slower microbial growth in breads containing FBSG compared to BF bread, WMF bread or bread containing BSG.

#### 3.2.7. In Vitro Starch Hydrolysis

**Figure 4:**
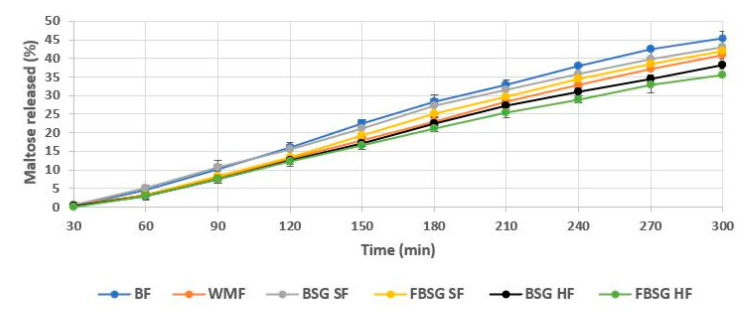
Comparison of the release of maltose over time from baked bread samples. BF and WMF denote “baker’s flour” and “wholemeal flour” breads, respectively. BSG and FBSG indicate “brewers’ spent grain” and “fermented brewers’ spent grain breads”, respectively. SF denotes “source of fibre addition level” and HF represents “high in fibre addition level”. Graphs show mean values of duplicate samples with standard deviations represented as error bars.

Starch digestibility was determined using an in vitro model system, and the release of reducing sugar (RSR) during digestion was investigated (Figure 4).

The highest release of reducing sugars over time was observed in BF bread, indicated by the highest slope (0.175 maltose released (%)/min). WMF bread showed a lower release of reducing sugars over time (slope: 0.157 maltose released (%)/min). The inclusion of BSG and FBSG decreased the starch digestibility of the breads, resulting in a lower release of sugars, particularly at high addition levels. FBSG HF breads showed the lowest degree of starch digestibility, leading to a slow sugar release with a slope of 0.137 maltose released (%)/min.

#### 3.2.8. Bread Ultrastructure

Scanning electron microscopy (SEM) was used to analyse changes in crumb structure with BSG and FBSG inclusion. Images of freeze-dried bread crumbs are illustrated in Figure 2.

The BF crumb (Figure 2A) displays partially gelatinised, porous starch granules embedded in a protein matrix. In contrast, the WMF bread crumb (Figure 2B) demonstrates a higher level of intact and more defined starch granules, indicating a lower level of starch gelatinisation.

BSG SF (Figure 2C) and FBSG SF (Figure 2D) exhibit similar findings to those observed in WMF crumb structure. A higher level of intact starch granules is evident. BSG HF (Figure 2E) and FBSG HF (Figure 2F) amplify the trends observed in SF formulations. In addition, a film associated with the starch granules occurred. Negligible differences were apparent in crumb structure in SEM images of BSG and FBSG formulations; however, FBSG HF (Figure 2F) images showed a smoother crumb matrix than BSG HF.

## 4. Discussion

The inclusion of BSG in bread is challenging, with higher inclusion levels leading to bread with significantly inferior quality than standard wheat bread. This study shows that processing of BSG using fermentation technology is a promising approach to aid in maintaining dough and bread quality. BSG and FBSG were included in bread formulations at two addition levels, “source of fibre” and “high in fibre”, with both the type of fibre ingredient and ingredient addition level significantly impacting dough quality and bread characteristics.

The gluten network strength and the gluten network development time are significant parameters in the breadmaking process to ensure a desirable dough and bread quality. The inclusion of BSG and FBSG, particularly at higher inclusion levels, resulted in a stronger network that developed faster than the BF control. The inclusion level, as well as the type of ingredient, significantly impacted gluten network strength (*p* < 0.001) and time to develop (*p* < 0.001). Fermentation alleviated the impact of BSG-derived ingredients on gluten network compared to unfermented BSG. Flours displaying a rapid gluten network aggregation and fast breakdown are regarded as poorer flours with weakened technical capacity [40,41,42]. BSG and FBSG ingredients contain a high amount of minerals (3.7%), proteins (31.4% and 32.4%, respectively) and fibres (42.6% and 49.4%, respectively), all of which can influence the strength and development time of gluten. Minerals can facilitate a charge screening effect, exposing apolar protein side-chains, causing greater hydrophobic interaction [43,44], and hence could result in a stronger network displayed by the higher torque. Furthermore, incorporating higher levels of protein could shift the balance of glutenin and gliadin present, leaning more towards a higher level of glutenin and increasing gluten strength [45]. Additionally, the inclusion of fibres has previously shown to enhance the kinetics of the gluten network [40], interact with the secondary structure of gluten proteins (primarily glutenin) and restrict hydration of the gluten network [46]. Arabinoxylans, the main fibre in BSG [2], have also been reported to be of particular hindrance to gluten formation [12,16,47,48,49]. The two peaks noted in Figure 1 at HF addition levels highlights potential secondary networks forming with the inclusion of BSG and FBSG at higher levels of addition. Previous investigations (Neylon et al. submitted for publication) revealed the presence of low molecular weight peptides present in BSG and FBSG, and with the introduction of charged amino acids from BSG [12], these conditions may facilitate the formation of secondary networks at a different time point to gluten formation [50]. The weaker gluten network and the more pronounced second peak in FBSG HF highlight a further modification to proteins post-fermentation. Proteins in FBSG undergo modifications during the fermentation process due to proteolysis and changes in pH with lactic acid production [51,52]. The weaker gluten network and more pronounced second peak observed could be linked with the acidic environment created with the introduction of the fermented ingredient. This could increase the positive charges present, which initially favours gluten network formation through the unfolding of the gluten proteins and enhancing hydrophobic interactions; however, the strong intermolecular forces cause a rapid breakdown of gluten and inhibits the formation of further bonds necessary for strong gluten formation [51,52,53]. The modified proteins/peptides in FBSG may also differ in charge and structure post fermentation, which may have induced further unfolding of proteins, exposing more hydrophobic regions and facilitating co-networking with gluten proteins via hydrophobic interactions to a greater extent [54].

As well as changes in gluten network development, differences in the viscoelastic behaviour of doughs were noted with BSG and FBSG inclusion, resulting in an increase in elastic parts in the dough. The damping factor was influenced by both the type of fibre ingredient (*p* < 0.001) and ingredient addition level (*p* < 0.001). Replacement of BF with BSG and FBSG ingredients reduced the amount of gluten and starch available within the dough matrix, resulting in a stiffer dough with greater resistance to deformation. However, replacement of BF with FBSG, predominantly at HF addition levels, led to a dough with more viscous parts than unfermented BSG, which emphasises the great potential of fermentation as a tool to functionalise BSG. As mentioned previously, the acids present in the fermented ingredient putatively induces an environment lower in pH, causing a weaker gluten network and reduces dough firmness [53] compared to unfermented BSG. In addition to this, the acidic environment can also enhance the proteolytic activity within the dough system, further reducing the elasticity and stiffness of the dough [51,53]. The damping factor also correlated positively with Hm (r = 0.9169, *p* < 0.01), highlighting that the more elastic properties of the doughs restricted their ability to rise and expand during proofing. This could be the reason for BSG HF and FBSG HF showing no dough rise (Hm = 0). Neither addition level (*p* < 0.345) nor type of fibre (*p* < 0.446) affected the volume of CO_2_ produced during proofing and did not differ significantly from BF. Hence, the yeast fermentation was not affected by the inclusion of BSG and FBSG, and the adverse effects observed during the dough’s rise are related to the dough rheology/structure imposed by the ingredients.

Dough rheology parameters such as Hm and the damping factor also significantly impacted bread quality characteristics. Both Hm and the damping factor correlated positively with specific volume (r = 0.96, *p* < 0.002, r = 0.82, *p* < 0.04, respectively). This highlights the significant effect of dough rheology on final bread volume. Specific volume was influenced by both ingredient addition level (*p* < 0.001) and the type of ingredient (*p* < 0.001). Fermentation of BSG led to an increase in specific volume, putatively due to the reductions observed in dough elasticity, which facilitated the dough’s expansion and rise. The reductions in specific volume with BSG and FBSG inclusion caused changes in crumb texture. Correlations between specific volume and crumb hardness (r = −0.85, *p* ≤ 0.03) as well as crumb resilience (r = 0.92, *p* ≤ 0.01) occurred, highlighting bread texture is dependent on the extent of the dough rise. Crumb hardness was influence by both ingredient type (*p* < 0.001) and level of addition (*p* < 0.001). Fermentation reduced crumb hardness, which was likely the result of the greater dough rise achieved during proofing (r = −0.92, *p* ≤ 0.01) combined with the more viscous nature of the dough with FBSG inclusion (r = −0.90, *p* ≤ 0.01) [53,55]. The impact of dough characteristics on crumb structure is highlighted by the positive correlation between cell diameter and Hm (r = 0.83, *p* ≤ 0.001) as well as the damping factor (r = 0.97, *p* ≤ 0.001). Bread crumb resilience is also an important bread quality parameter and was affected mainly by ingredient addition level (*p* < 0.001) rather than the type of fibre ingredient. Both BSG-derived ingredients weakened crumb resilience, putatively due to the changes observed in the gluten network formation, which impacts gluten quality and the adverse effects noted in dough rheological properties.

Apart from the gluten network and the viscoelastic properties of the dough, changes in the viscosity of the formulations during heating also influenced bread quality. As a general trend, replacement of BF with more fibrous ingredients resulted in a reduction in peak viscosities, likely due to the reduction in the overall starch content, as well as the increase in competition for hydration by the fibre and protein fraction of BSG and FBSG [47,56,57]. Both ingredient type (*p* < 0.006) and addition level (*p* < 0.001) influenced the evaluated pasting parameters. Peak viscosity represents the highest viscosity reached during heating and correlated positively with specific volume (r = 0.88, *p* ≤ 0.02). Higher degrees of starch swelling facilitate a greater expansion in starch granules, which aids in achieving a higher specific volume [58]. The incorporation of FBSG resulted in a higher peak viscosity than BSG, putatively due to the slightly lower amount of BF replacement, resulting in a higher total amount of starch susceptible for pasting. Furthermore, as mentioned before, the inclusion of BSG-derived ingredients leads to an increase in competition for water with starch, resulting in a lower degree of starch gelatinisation. This can be observed in the micrographs, showing higher amounts of intact starch granules in the crumb of high in fibre BSG/FBSG breads.

After the peak, a breakdown of viscosity occurs due to starch leaching, resulting in the trough viscosity before cooling. FBSG showed a higher breakdown than BSG, most likely due to the higher amylase activity in the FBSG ingredient, resulting in reduced starch granule rigidity and enhancing sensitivity to deformation [59]. The final viscosity indicates the degree of retrogradation of the system during cooling. BSG and FBSG caused a lower final viscosity, most likely due to the higher amounts of fibre present, which interrupts the realignment of the macromolecular matrix during the cooling process through physical disruption, obstruction of secondary forces and sterical hindrance [56]. Furthermore, the significantly lower final viscosities observed in formulations including FBSG may be linked with the higher amylase activity of the FBSG ingredient, decreasing the degree of retrogradation [60,61,62]. Amylases partially degrade amylopectin and amylose, negatively influencing their rearrangement during retrogradation [63]. Moreover, the inclusion of FBSG introduces lactic acid to the system, which has previously shown to restrict starch retrogradation [64] and increase solubility of amylopectin, which may further inhibit the realignment process [65]. However, higher bread staling rates occurred in breads including FBSG compared to BSG, which is putatively due to the lower replacement level of BF by FBSG. This led to a higher total starch level in formulations containing FBSG compared to BSG. Furthermore, it needs to be mentioned that the dense crumb structure caused high crumb hardness already after baking. Hence, the increase in hardness over time was less pronounced.

The denser crumbs might also be the reason for the extended microbial shelf life observed in high-fibre breads, with a denser crumb potentially restricting the aeration needed for microbial growth [66]. Furthermore, the inclusion of FBSG appears to exhibit some anti-microbial properties and slows the kinetics of microbial growth overtime compared to BSG. Sourdough technology using a variety of different lactic acid bacteria has previously shown to induce an anti-microbial effect, which has been attributed to the combined acidification and the synergistic effect of the various anti-microbial metabolites produced during lactic acid bacteria fermentation [67,68].

Besides extending microbial shelf life, bread fortified with BSG-derived ingredients resulted in a lower sugar release during starch digestion. This likely occurred mainly due to the reduction of available carbohydrates caused by wheat flour replacement. Furthermore, the incorporation of fibre and protein is known to restrict the extent of enzyme hydrolysis [69,70]. More densely packed food structures can also be limiting factors to enzyme activity [71,72], and the dense crumb structure of BSG and FBSG may further inhibit enzyme-substrate affinity. The micrographs of high fibre breads including BSG or FBSG also revealed a film in association with the starch granules, which could be the product of potential protein–starch–fibre interactions [48,69,71,73]. This film could act as a further barrier for enzyme hydrolysis, leading to a lower sugar release [69,71,74,75]. Fermentation of BSG resulted in a lower release of sugars during in vitro starch digestions. This may be attributed to the lactic acid produced during fermentation which creates a more acidic environment and hinders starch hydrolysis [76,77,78,79]. A study by Östman et al. [80] proposed a potential mechanism for this, indicating lactic acid present during heat treatment induces interactions between starch and gluten and limits the bioavailability of starch for enzyme hydrolysis.

## 5. Conclusions

Rejuvenating BSG for bakery application, particularly breadmaking, is challenging because of its high impact on dough rheology and bread quality characteristics. Hence, new approaches which involve processing are needed. The results from this study highlight the great potential of fermentation as a tool to functionalise BSG and turn it into a food ingredient, which elevates the nutritional value of bread by increasing protein and fibre content and simultaneously ensuring higher bread quality. The inclusion of FBSG reduced dough stiffness and affected the gluten network formation to a lesser extend compared to unfermented BSG. These changes in the dough system positively enhanced bread techno-functional properties, resulting in an increase in bread specific volume and reduction in crumb hardness. In addition to the improved bread quality, fermentation of BSG resulted in an ingredient that prolonged microbial shelf life and reduced the staling of bread. Furthermore, the fermentation of BSG can positively enhance the nutritional value of the ingredient by decreasing the release of sugar during digestion. Further investigations related to the optimisation of the baking process by adjusting the mixing process, for example, would be of great importance. Moreover, additional baking aids, such as dough improvers, might ameliorate the dough rheology and result in higher bread quality. This work highlights the excellent potential of fermentation technology as a processing aid that could further valorise BSG as a food ingredient in the future.

## Figures and Tables

**Table 1:** Compositional analysis of baker’s flour (BF), wholemeal flour (WMF), brewers’ spent grain (BSG) and fermented brewers’ spent grain (FBSG) flour ingredients in g/100 g.

	BF	WMF	BSG	FBSG
Protein	12.9	11.4	31.4	32.4
Moisture	12.6	12.0	4.7	5.0
Fat	0.86	1.6	10.3	6.5
Ash	0.70	1.3	3.7	3.7
Total Carbohydrate	72.9	73.7	49.9	52.37
Of which dietary fibre	3.1	7.1	42.6	49.4
α-amylase (cu/g) *	0.12 ± 0.01	0.12 ± 0.02	0.12 ± 0.00	0.24 ± 0.00

* Cu/g = ceralpha units/g flour. (One ceralpha unit represents the amount of enzyme needed to release 1 µmol of *p*-nitrophenol per min at 40 °C (in the presence of excess α-glucosidase) [35]).

**Table 2:** Bread recipes expressed as % based on flour + fibre ingredient (=100%). BF and WMF represents Baker’s flour and Wholemeal Flour, respectively. SF and HF represent “source of fibre addition level” and “high in fibre addition level”, respectively. BSG and FBSG denotes “brewers’ spent grain” and “fermented brewers’ spent grain”, respectively.

Ingredient	BF	WMF	BSG (SF)	FBSG (SF)	BSG (HF)	FBSG (HF)
Baker’s flour	100	-	95.0	96.0	82.0	85.0
Wholemeal	-	100	-	-	-	-
Fibre ingredient	-	-	5.0	4.0	18.0	15.0
Salt	1.2	1.2	1.2	1.2	1.2	1.2
Sugar	2.0	2.0	2.0	2.0	2.0	2.0
Sunflower oil	3.2	3.2	3.2	3.2	3.2	3.2
Dry Yeast	2.0	2.0	2.0	2.0	2.0	2.0
Water	57.3	59.3	61.6	60.1	68.6	66.9

- represents “not applicable”.

**Table 3:** Results from the effect of brewers’ spent grain (BSG) and fermented brewers’ spent grain (FBSG) addition at source of fibre (SF) and high in fibre (HF) inclusion levels on farinograph water absorption capacities, gluten aggregation properties, starch pasting behaviour, dough rheology properties, and fermentation capacity. BF and WMF represent results obtained for baker’s flour and wholemeal flour controls, respectively. The values provided represent the mean ± the standard deviation. Values that share the same letter in the same row do not differ significantly.

	BF	WMF	BSG (SF)	FBSG (SF)	BSG (HF)	FBSG (HF)
Farinograph						
Water Absorption %	57.30 ± 0.2 ^f^	59.33 ± 0.15 ^e^	61.60 ± 0.1 ^c^	60.10 ± 0.1 ^d^	68.60 ± 0.35 ^a^	66.93 ± 0.31 ^b^
GlutoPeak						
Peak Max Time (s)	48.67 ± 1.53 ^b^	141.33 ± 15.18 ^a^	47.67 ± 1.15 ^b^	40.33 ± 0.58 ^c^	15.67 ± 2.08 ^d^	14.67 ± 0.58 ^d^
Torque Max (BU)	71.67 ± 0.58 ^c^	29.0 ± 1.0 ^e^	57.33 ± 0.58 ^d^	60.67 ± 0.58 ^d^	100.0 ± 2.0 ^a^	84.0 ± 2.65 ^b^
Rapid Visco Analyser						
Peak Viscosity (cP)	1007.67 ± 14.57 ^a^	591.33 ± 30.85 ^e^	911.0 ± 15.13 ^b^	900.66 ± 13.05 ^b^	701.33 ± 5.13 ^d^	760.67 ± 10.69 ^c^
Final Viscosity (cP)	1327.33 ± 26.58 ^a^	1371.67 ± 27.61 ^a^	1229.69 ± 23.69 ^b^	1038.0 ± 11.79 ^c^	992.67 ± 8.33 ^c^	643.0 ± 6.24 ^d^
Trough (cP)	607.0 ± 15.52 ^a^	486.0 ± 25.71 ^c^	543.67 ± 11.59 ^b^	486.67 ± 13.58 ^c^	438.67 ± 3.79 ^d^	321.33 ± 10.26 ^e^
Breakdown (cP)	400.67 ± 1.15 ^b^	105.33 ± 10.96 ^e^	367.33 ± 4.04 ^c^	414.0 ± 3.61 ^a,b^	262.67 ± 1.53 ^d^	439.33 ± 7.23 ^a^
Rheology						
Damping factor	0.368 ± 0.015 ^a^	0.341 ± 0.007 ^b^	0.331 ± 0.004 ^b^	0.330 ± 0.004 ^b^	0.264 ± 0.005 ^d^	0.280 ± 0.003 ^c^
Rheofermentometer						
Height max (mm)	53.33 ± 1.7 ^a^	20.3 ± 0.44 ^c^	35.03 ± 0.6 ^b^	38.4 ± 3.48 ^b^	0 ± 0 ^d^	0 ± 0 ^d^
Total Vol CO_2_ (mL)	2159.3 ± 132.03 ^a^	2237.7 ± 71.93 ^a^	2139.6 ± 118.5 ^a^	2124.0 ± 62.81 ^a^	2114.6 ± 76.8 ^a^	2047.6 ± 89.51 ^a^
CO_2_ retention coefficient (%)	98.73 ± 0.74 ^a,c^	99.60 ± 0.10 ^a,b^	99.36 ± 0.15 ^a,c^	99.20 ± 0.10 ^c^	99.73 ± 0.06 ^a^	99.70 ± 0.10 ^a^

**Table 4:** Results from analysis of the techno-functional properties of bread with inclusion of brewers’ spent grain (BSG) and fermented brewers’ spent grain (FBSG) at source of fibre (SF) and high in fibre (HF) addition levels. BF and WMF represents results obtained from baker’s flour and wholemeal flour breads, respectively. The values shown represent the mean ± the standard deviation. Values which have the same letter in the same row do not differ significantly.

	BF	WMF	BSG (SF)	FBSG (SF)	BSG (HF)	FBSG (HF)
Fibre Content (g/100)	2.10	4.76	3.32	3.27	6.41	6.37
Digestible Starch content of breads (g/100)	38.74 ± 0.55 ^a^	33.81 ± 0.21 ^c^	37.03 ± 0.97 ^a^	35.70 ± 0.97 ^b^	29.38 ± 0.74 ^d^	31.37 ± 0.43 ^c^
Bake loss (%)	15.04 ± 0.53 ^a^	12.03 ± 0.51 ^c^	12.89 ± 0.40 ^b^	13.00 ± 0.69 ^b^	10.23 ± 0.26 ^d^	10.47 ± 0.40 ^d^
Specific Volume (mL/g)	5.49 ± 0.11 ^a^	2.07 ± 0.11 ^d^	3.49 ± 0.13 ^c^	3.86 ± 0.21 ^b^	1.45 ± 0.05 ^f^	1.69 ± 0.03 ^e^
Slice Area (mm^2^)	11654 ± 361 ^a^	5127 ± 361 ^d^	8060 ± 313 ^c^	8788 ± 471 ^b^	4776 ± 278 ^e^	5214 ± 234 ^d^
Number of cells	6472 ± 282 ^a^	3250 ± 168 ^c^	5556 ± 225 ^b^	5593 ± 246 ^b^	5483 ± 426 ^b^	5441 ± 387 ^b^
Cell diameter (mm)	2.28 ± 0.07 ^a^	2.18 ± 0.14 ^b^	1.72 ± 0.08 ^d^	1.86 ± 0.10 ^c^	1.09 ± 0.06 ^f^	1.15 ± 0.07 ^e^
Bread Texture						
Hardness T2 (N)	2.99 ± 0.36 ^f^	30.13 ± 6.15 ^c^	10.91 ± 1.32 ^d^	7.91 ± 1.31 ^e^	79.22 ± 5.88 ^a^	47.24 ± 3.97 ^b^
Resilience (T2)	0.49 ± 0.02 ^a^	0.41 ± 0.02 ^c^	0.46 ± 0.02 ^b^	0.47 ± 0.02 ^b^	0.34 ± 0.02 ^d^	0.34 ± 0.02 ^d^
Stale rate	2.20 + 0.48 ^a^	0.95 ± 0.46 ^c,d^	1.22 ± 0.36 ^b,c^	1.59 ± 0.34 ^b^	0.72 ± 0.24 ^d^	1.04 ± 0.21 ^c,d^
Colour						
ΔE Crust (Baker’s Flour)	-	-	8.90 ± 1.03 ^b^	8.39 ± 1.30 ^b^	9.26 ± 1.25 ^b^	11.99 ± 1.25 ^a^
ΔE Crust (Wholemeal flour)	-	-	20.91 ± 1.11 ^a^	20.59 ± 1.07 ^a^	12.85 ± 0.96 ^b^	10.52 ± 0.71 ^c^
ΔE Crumb (Baker’s Flour)	-	-	14.64 ± 1.16 ^c^	15.24 ± 0.95 ^c^	29.18 ± 0.55 ^a^	28.31 ± 0.86 ^b^
ΔE Crumb (Wholemeal flour)	-	-	8.54 ± 0.66 ^d^	9.17 ± 0.75 ^c^	11.61 ± 0.77 ^a^	10.50 ± 0.75 ^b^
Water Activity	0.95 ± 0.01 ^b^	0.96 ± 0.01 ^a,b^	0.97 ± 0.01 ^a^	0.97 ± 0.01 ^a^	0.97 ± 0.01 ^a^	0.97 ± 0.01 ^a^

- represents “not applicable”.
